# Weight loss outcomes with tirzepatide in women with and without self-reported polycystic ovary syndrome

**DOI:** 10.1210/jendso/bvag177

**Published:** 2026-08-19

**Authors:** Ashley Kieran Clift, Dan Reisel, Hans Johnson, Naveed Sattar, David R Huang

**Affiliations:** Department of Clinical Research, Voy (Menwell Ltd), London SE1 9HZ, UK; Department of Surgery & Cancer, Imperial College London, London W12 0NN, UK; Department of Clinical Research, Voy (Menwell Ltd), London SE1 9HZ, UK; EGA Institute for Women's Health, University College London, London WC1E 6DE, UK; Department of Clinical Research, Voy (Menwell Ltd), London SE1 9HZ, UK; Department of Digital Health and Care, University of Bristol, Bristol BS1 5DD, UK; BHF Glasgow Cardiovascular Research Centre, University of Glasgow, Glasgow G12 8TD, UK; Department of Clinical Research, Voy (Menwell Ltd), London SE1 9HZ, UK

**Keywords:** obesity, polycystic ovary syndrome, tirzepatide, digital weight loss service

## Abstract

**Context:**

Weight management is key to managing polycystic ovary syndrome (PCOS) and overweight/obesity but is challenging for many. Evidence regarding tirzepatide's efficacy in women with polycystic ovary syndrome is limited.

**Objectives:**

To quantify the effectiveness of tirzepatide for weight loss in women with (self-reported) polycystic ovary syndrome and overweight/obesity compared with women not living with PCOS and to explore associations between engagement with nonpharmacological digital adjuncts and weight loss.

**Methods:**

Retrospective open-cohort study in a digital weight management service in the United Kingdom. All women (aged 18+ years) with overweight/obesity prescribed tirzepatide for weight management between February 2024 and January 2025 were included. Percentage weight change from baseline was calculated in women reaching specific follow-up time points, stratified by PCOS status. Kaplan-Meier methods estimated cumulative probabilities of attaining total body weight loss thresholds.

**Results:**

The cohort comprised 54 114 women of whom 4241 (7.84%) women had PCOS. At 10 months, the mean weight change in women with PCOS (n = 40) was −19.40% (95% confidence interval [CI], −22.38% to −16.42%); this was not significantly different from women without PCOS (n = 397; mean weight change, −18.74%; 95% CI, −19.67% to −17.81%). A total of 57.66% of women with PCOS lost ≥20% total body weight (95% CI, 47.44% to 68.29%) by 10 months. In women living with PCOS, digitally engaged women lost 3.42% more weight in absolute terms (95% CI, 1.71% to 5.12%; *P* < .001) by 10 months.

**Conclusion:**

Tirzepatide may offer a highly effective therapeutic option for women with PCOS and overweight/obesity in whom weight loss is a key goal but a persistent challenge.

Polycystic ovary syndrome (PCOS) is a common endocrine condition affecting 10% to 15% of women of reproductive age and is characterized by hyperandrogenism, ovulatory dysfunction, and polycystic ovarian morphology on ultrasound (1-3). Women with PCOS often exhibit a spectrum of metabolic disturbances, including insulin resistance, obesity, dyslipidemia, and an elevated risk of type 2 diabetes mellitus and cardiovascular disease, all of which contribute to long-term morbidity (3). Even modest weight loss (≥5% of body weight) can restore ovulatory cycles, improve insulin sensitivity, and thus reduce hyperinsulinemia and, in turn, improve androgen levels and lipid profiles (1, 4, 5). Attaining ≥5% weight loss is therefore a key component of management for women with PCOS and overweight/obesity (6). However, attaining and sustaining such weight loss through lifestyle modification alone is challenging for many patients given the constancy of the obesogenic environment.

Current therapeutic strategies for PCOS include lifestyle modification, insulin sensitizers such as metformin, antiandrogens, and, in selected cases, bariatric surgery (7, 8). However, medical interventions frequently fail to produce durable improvements in weight and metabolic control, particularly in women with obesity and PCOS (9). Glucagon-like peptide-1 (GLP-1) receptor agonists (RAs) have emerged as promising agents in this context. In women with PCOS, liraglutide and exenatide have demonstrated superior reductions in body mass index (BMI), waist circumference, and insulin resistance compared with metformin alone, as well as improvements in menstrual regularity and androgen profiles (10). More recently, tirzepatide, a dual agonist of GLP-1 and glucose-dependent insulinotropic polypeptide receptors, has outperformed selective GLP-1 RAs such as semaglutide in achieving weight loss and metabolic benefits in people with obesity (11).

Despite compelling efficacy in observational and randomized studies, data on tirzepatide's effects specifically in women with PCOS are limited and largely extrapolated from animal models or “overall” results from obesity trials (12-15). A recent narrative review underscored the lack of studies analyzing outcomes with antiobesity medications specifically in PCOS (14), and the pharmaceutical company that developed tirzepatide has not sponsored any trials evaluating the effectiveness of tirzepatide in this group (16). This is notable as treatment efficacy can be patterned by metabolic comorbidities; for example, antiobesity pharmacotherapies are less effective in people with type 2 diabetes (17). In this context of mechanistic promise, comorbidity-associated patterning and evidence limitations, it is important to characterize the effectiveness of tirzepatide, specifically in women with PCOS, to inform modern treatment recommendations and guide future trials for this high-risk population with high potential for benefit.

To address this evidence gap, the present study evaluated the real-world effectiveness of tirzepatide in a cohort of women treated at a digital weight management service, stratified by their baseline self-reported PCOS status. This study then explored the association between digital engagement and weight loss outcomes in groups defined by their self-reported PCOS status (reported vs not).

## Materials and methods

### Study design, setting, and participants

We conducted a retrospective open cohort study of women who were prescribed tirzepatide as part of a digital weight management service in the United Kingdom (Voy, operated by Menwell Ltd). Study participants were women (self-reported female sex) aged 18 years or older at the date of tirzepatide prescription, with a baseline BMI of 30 kg/m^2^ or higher, or a baseline BMI of 27 kg/m^2^ with at least 1 obesity-related comorbidity. In this study, PCOS status was based on self-report via a baseline questionnaire.

The cohort period ran from February 1, 2024, to January 1, 2025. All women commencing treatment with tirzepatide at any point within this timeframe entered the cohort. Patients contributed follow-up from the date of first prescription until the earliest of the following: reached 10 months follow-up, reached cohort study end date, or stopped using tirzepatide (eg, reached target weight or discontinued using the service). In addition to obesity pharmacotherapy, access to the Voy app was provided through which individuals booked lifestyle coaching sessions (individual or group), tracked weight, and engaged with informational materials. These nonpharmacological services were provided for free, and their use was optional.

Women were advised that tirzepatide should be avoided during pregnancy and breastfeeding, that the medication must be stopped if trying to conceive or become pregnant, and that they should wait at least 1 month before trying to conceive after stopping tirzepatide.

### Outcome and variable definitions

The primary outcome was percentage weight change from baseline in women living with self-reported PCOS compared with women without self-reported PCOS. Secondary outcomes were (1) the proportion of women attaining preset thresholds of total body weight loss by 10 months (≥5%, ≥10%, ≥15%, and ≥20%) and (2) percentage weight change over 10 months in women who engaged with nonpharmacological aspects of the weight management service compared with those who did not.

For the primary outcome, weight data recorded by individuals and provided via the Voy app were used; it was mandatory to provide a weight measurement at least once a month for continued tirzepatide prescriptions. Therefore, for example, a woman who used tirzepatide for 4 months would have weight data for baseline and at least monthly measurements thereafter for the 4 months of follow-up. Weight change from baseline was calculated for each month of follow-up; where an individual contributed multiple weight measurements in a 30-day period, the latest was used. For the secondary outcome, digital engagement was defined as meeting all 3 of the following criteria: attended at least 1 lifestyle coaching session, tracked weight at least weekly on average, and accessed the information materials on the Voy app at least once. This was defined a priori by the study team (18). Data regarding baseline age and self-reported comorbidities (PCOS, diabetes mellitus, hypertension, high cholesterol, metabolic dysfunction-associated fatty liver disease) were also collected during service enrollment.

Based on clinical judgement and previous literature (19), individuals' measurements were excluded from the study if they had implausible values: baseline BMI>100 kg/m^2^, a BMI change velocity exceeding ±4 kg/m^2^ from 1 month to the next, height <1.3 meters or height >2.2 meters, or recorded weight over 300 kg.

### Statistical analysis

Descriptive statistics summarized baseline characteristics with medians and interquartile ranges. Due to the open cohort design, there was variation in the length of individuals' follow-up. Women could have commenced tirzepatide at any point during the open cohort period (and therefore have a shorter follow-up duration due to censoring) or could have discontinued the medication.

For the primary outcome, we performed a “completer analysis”, wherein the mean weight changes with 95% confidence intervals (CIs) at follow-up intervals (2, 4, 6, 8, and 10 months) were calculated using data from the subpopulation of women reaching that time point on treatment. For example, mean percentage weight change at 6 months was calculated using data from the subgroup of women reaching 6 months of follow-up on tirzepatide treatment. Kaplan-Meier methods were used to estimate the cumulative incidence of attaining specific thresholds of total body weight loss over time (≥5%, ≥10%, ≥15%, and ≥20%) with 95% CIs.

To explore associations between self-reported PCOS status, digital engagement, and weight change over time, we utilized adjusted mixed models for repeated measures. In each of these models, percentage weight change from baseline was the dependent variable, with a 3-way interaction between a binary variable denoting engagement status, a binary variable denoting self-reported PCOS status, and a categorical variable for month. These models were adjusted for baseline weight, age, and self-reported comorbidities (ie, diabetes, hypertension, high cholesterol, and metabolic dysfunction-associated liver disease). The marginal means and corresponding 95% CIs were extracted.

Data were complete for the outcome and all covariates (ie, no missing data imputation was required). R statistical software (v4.4.2) and STATA V18 were used for data processing and analysis. A 2-sided *P*-value <.01 was considered significant. The STROBE checklist was used for study reporting (20).

### Ethical approval

This retrospective study used anonymized data collected routinely during standard care pathways. Consent for the original collection of data was obtained by patients' agreement on service enrollment that anonymized data could be used for service evaluation, improvement, and audit purposes. This study was approved by the University College London Research Ethics Committee (ID: 2025-0906-775).

## Results

### Study cohort characteristics

Of 66 846 individuals enrolling in the tirzepatide-based digital weight management program during the cohort window, 54 114 (80.95%) were women and formed the study cohort. Of these, 4241 (7.84%) had self-reported PCOS at baseline. Summary characteristics of the cohort and subcohorts are summarized in Table 1. The mean follow-up duration was 2.28 months. At the point of data extraction, 2126 women living with self-reported PCOS (50.13%) were still actively on treatment with tirzepatide, and 22 936 of the women without self-reported PCOS were on active treatment (45.99%) [see Figs. S1 and S2 (21) for flow charts detailing follow-up and censoring over time for both groups].

**Table 1:** Summary characteristics of the study cohort

	Women living with overweight/obesity and self-reported PCOS			Women living with overweight/obesity but without self-reported PCOS		
Parameter	Subcohort	Digitally nonengaged	Digitally engaged	Subcohort	Digitally nonengaged	Digitally engaged
Number of women	4241	3641 (85.85)	600 (14.15)	49 873	44 757 (89.74)	5116 (10.26)
Age, years, median (IQR)	34 (29-40)	34 (29-40)	36 (31-43)	42 (34-52)	42 (33-51)	45 (37-54)
BMI, kg/m^2^, median (IQR)	35.56 (31.22-40.92)	35.20 (30.95-40.33)	37.91 (33.06- 43.14)	33.91 (31.31-38.10)	33.82 (31.25-37.95)	35.16 (31.87-39.69)
Diabetes mellitus	212 (5.00)	170 (4.67)	42 (7.00)	1333 (2.67)	1161 (2.59)	172 (3.36)
Hypertension	349 (8.23)	293 (8.05)	56 (9.33)	4266 (8.55)	3648 (8.15)	618 (12.08)
High cholesterol	265 (6.25)	222 (6.10)	43 (7.17)	2798 (5.61)	2404 (5.37)	394 (7.70)
Metabolic dysfunction-associated fatty liver disease	203 (4.79)	170 (4.67)	33 (5.50)	761 (1.53)	611 (1.37)	150 (2.93)

Data are given as n (%) unless otherwise noted. Parameters are given at baseline.

Abbreviations: IQR, interquartile range; PCOS, polycystic ovary syndrome.

### Characterizing weight loss outcomes in women prescribed tirzepatide by PCOS status

Of the 49 873 women without self-reported PCOS who commenced treatment, 5116 (10.26%) reached 6-month follow-up, and 397 (0.79%) reached 10-month follow-up on tirzepatide. Of the 4241 women with self-reported PCOS who commenced treatment, 472 reached 6-month follow-up (11.13%), and 40 reached 10 months of follow-up (1.18%) on tirzepatide. The mean percentage weight change at 2-month intervals is summarized in Table 2 with the numbers of women contributing follow-up to the cohort at these time points. Given the time-dependent effect of GLP-1 RAs on weight loss, in this section, we mostly discuss results at the latest follow-up point for the cohort, but more granular results are available in tables and figures (as noted in the following discussion).

**Table 2:** Percentage weight change during follow-up with 95% confidence intervals and numbers of women contributing follow-up at these intervals

	Women living with overweight/obesity and self-reported PCOS		Women living with overweight/obesity but without self-reported PCOS	
Month of follow-up	Women contributing follow-up at time point, n (% of subcohort)	Weight change in women reaching follow-up time, mean % (95% CI)	Women contributing follow-up at time point, n (% of subcohort)	Weight change in women reaching follow-up time, mean % (95% CI)
2	1581 (37.28)	−6.33 (−6.52 to −6.14)	16 242 (32.57)	−6.65 (−6.71 to −6.59)
4	898 (21.17)	−11.18 (−11.60 to −10.76)	9068 (18.18)	−12.06 (−12.20 to −11.93)
6	472 (11.13)	−14.91 (−15.63 to −14.19)	5116 (10.26)	−15.37 (−15.58 to −15.12)
8	173 (4.07)	−17.09 (−18.53 to −15.64)	1777 (3.56)	−17.34 (−17.79 to −16.90)
10	40 (0.94)	−19.40 (−22.38 to −16.42)	397 (0.80)	−18.74 (−19.67 to −17.81)

In women living with self-reported PCOS completing 10 months of follow-up on tirzepatide treatment (n = 40), the mean percentage weight change was −19.40% (95% CI, −22.38% to −16.42%). This was not significantly different from women without self-reported PCOS (mean percentage weight loss −18.74%; 95% CI, 19.67% to −17.81%; *P* = .68).

**Figure 1:**
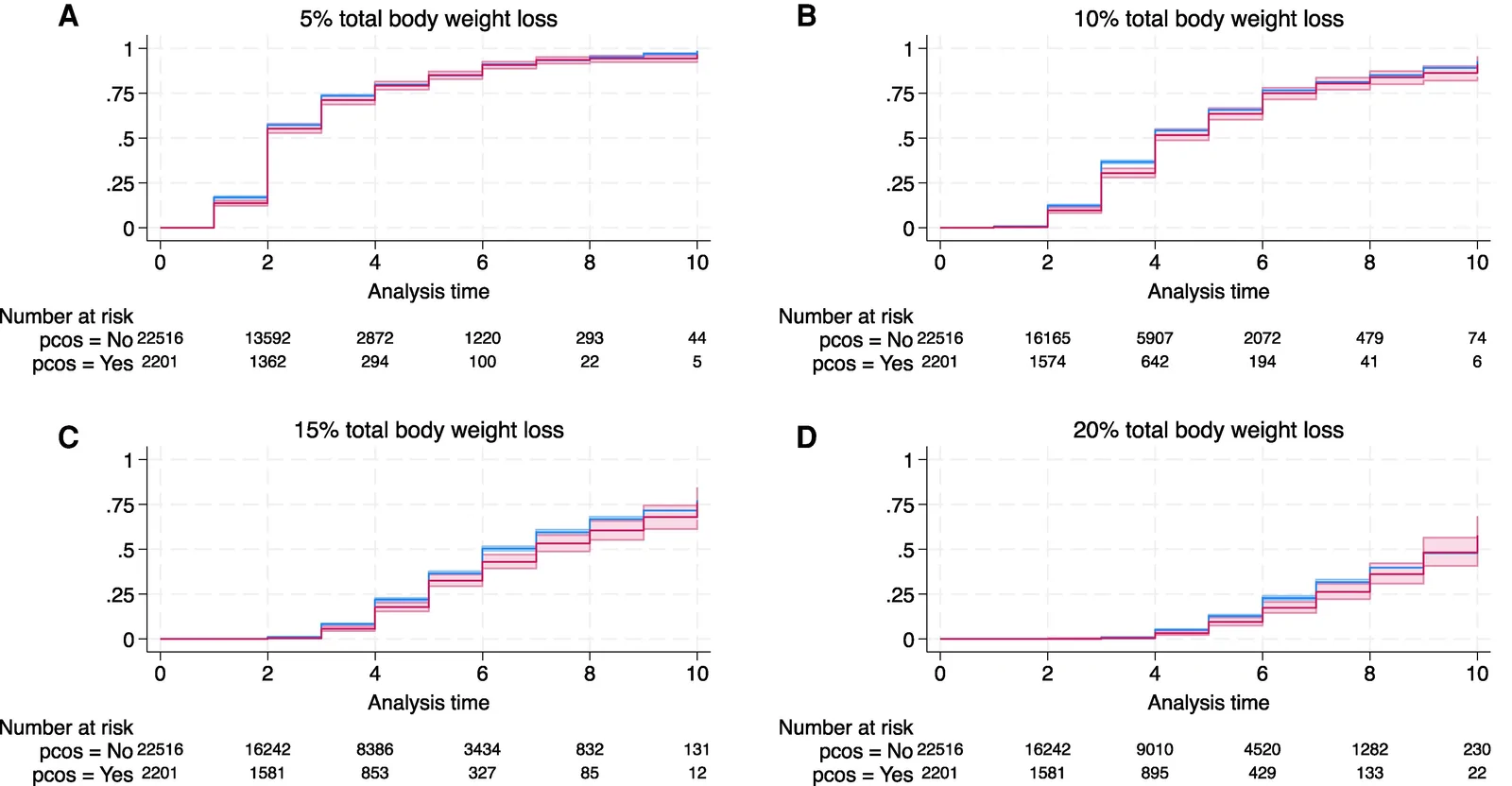
Kaplan-Meier estimates of cumulative probability of attaining thresholds of total body weight loss during follow-up in the cohort, stratified by self-reported polycystic ovary syndrome status; (A) 5% total body weight loss; (B) 10% total body weight loss; (C) 15% total body weight loss; (D) 20% total body weight loss.

On Kaplan-Meier analysis, by 10 months, 96.58% of women with self-reported PCOS lost ≥5% total body weight (95% CI, 93.11% to 98.59%), 90.80% lost ≥10% total body weight (95% CI, 83.94% to 95.55%), 75.96% lost ≥15% total body weight (95% CI, 66.30% to 84.55%), and 57.66% lost ≥20% total body weight (95% CI, 47.44% to 68.29%). These were not significantly different from women without self-reported PCOS (log-rank tests, all *P*s > .05) (Fig. 1).

### Exploring associations between digital engagement and weight loss outcomes

**Figure 2:**
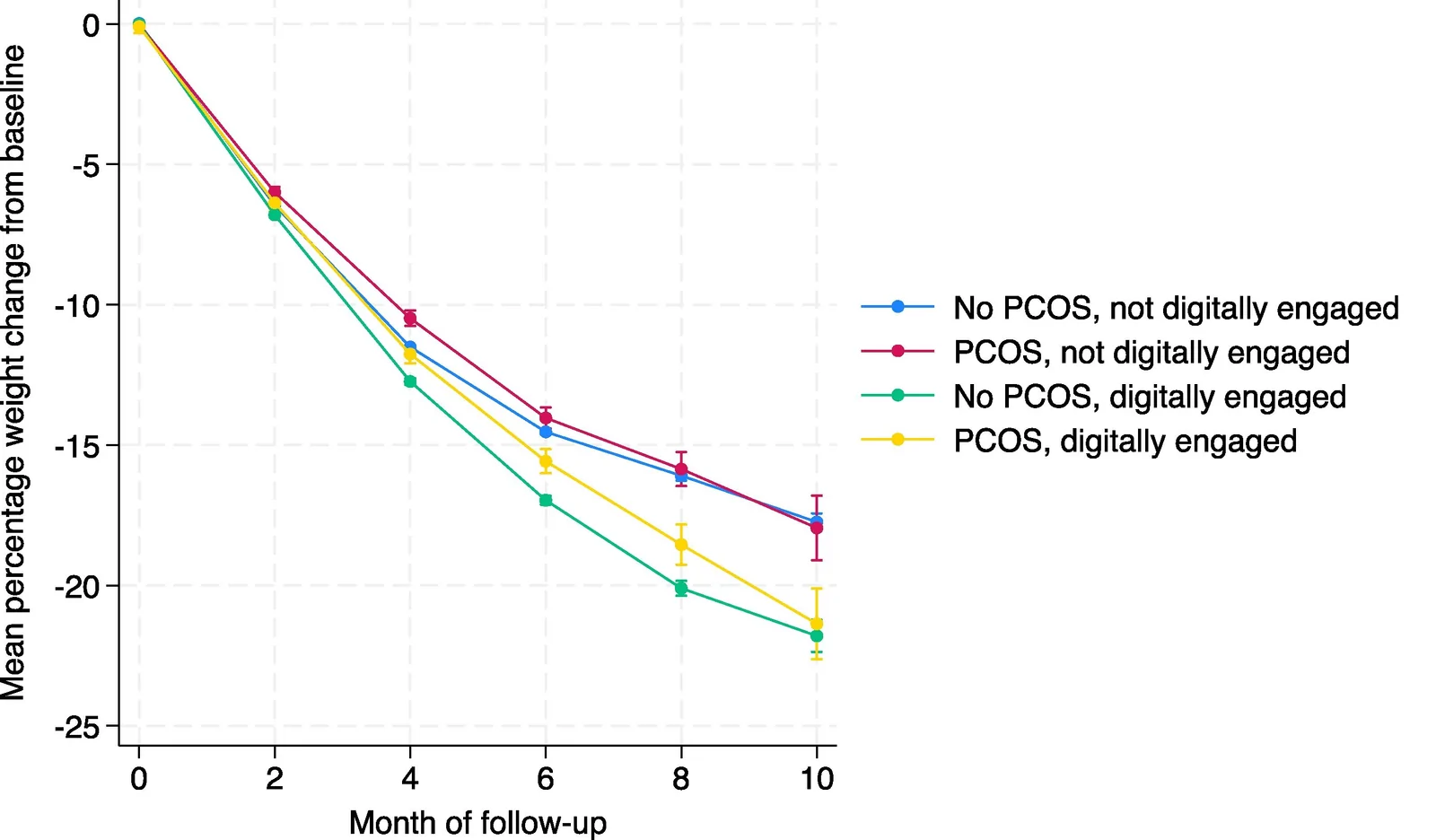
Percentage weight change from baseline in women living with overweight/obesity treated with tirzepatide stratified by both self-reported polycystic ovary syndrome (PCOS) status and digital engagement status. Average percentage weight loss (with 95% confidence intervals) at 2-month intervals was calculated from coefficients from linear mixed models for repeated measures. Models were adjusted for baseline weight, age, and other [non-PCOS] comorbidities.

Six hundred women with self-reported PCOS met the 3 criteria for digital engagement (14.15% of the subcohort) during follow-up. In women without self-reported PCOS, 5116 (10.26% of the subcohort) met the 3 criteria. Weight loss trajectories between digitally engaged and nonengaged groups appeared to diverge by month 6, with the engaged groups losing significantly more weight at each time point thereafter (Fig. 2). At month 10, the mean weight change in women with self-reported PCOS who were digitally engaged was −21.27% (95% CI, −22.63% to −20.11%); this was not significantly different from the mean weight change in women without self-reported PCOS who were digitally engaged (mean −21.80%; 95% CI, −22.37% to −21.23%). However, for both subcohorts, weight loss was significantly greater in the digitally engaged groups.

In women with self-reported PCOS, there was a 3.42% (95% CI, 1.71% to 5.12%; *P* < .001) greater weight loss in absolute terms associated with digital engagement. In women without self-reported PCOS, there was a 4.06% (95% CI, 3.41% to 4.70%; *P* < .001) greater weight loss in absolute terms associated with digital engagement.

## Discussion

This retrospective cohort study provides evidence regarding the potential weight loss effects of tirzepatide in women living with self-reported PCOS and overweight or obesity. The apparent effectiveness of the medication did not appear to be patterned by self-reported PCOS status. By 10 months of treatment, the mean weight reduction approached 20% for those women who reached this point and stayed on the medication. Furthermore, we estimated that nearly all women living with self-reported PCOS achieved the oft-promulgated target of ≥5% total body weight loss (3, 6, 15), and the vast majority achieved ≥10% total body weight loss. These findings suggest that tirzepatide may offer a highly effective therapeutic option for weight loss in women with PCOS and overweight/obesity, a numerically large population in which weight loss is a key goal due to multiple direct and indirect benefits but remains a persistent challenge.

The magnitude of weight loss observed in this cohort is broadly similar to that reported in clinical trials of tirzepatide for individuals with obesity or type 2 diabetes (14, 22). This is clinically relevant, as weight loss of ≥5% is associated with improvements in insulin resistance, ovulatory function, and cardiometabolic risk markers in women with PCOS (9). The study also observed that in addition to tirzepatide's pharmacological effects, digital engagement with nonpharmacological support functions was associated with a larger degree of weight loss over the course of 10 months. Participants who regularly used the digital platform (ie, attending lifestyle-coaching sessions, logging weight weekly, and interacting with the app) achieved an additional 3.42% weight loss in absolute terms. Self-monitoring, personalized feedback, goal-setting, and peer support have been demonstrated elsewhere to boost adherence and augment outcomes in obesity management (23, 24), and this could be applicable to the cohort studied. That said, we accept there may be a bias in women who are motivated to use this app being more motivated in general.

Evidence pertaining to the use of tirzepatide in women with PCOS is markedly limited (10, 15). In one single-center, nonrandomized retrospective study available as an abstract, 30 nondiabetic women with PCOS were treated with combined metformin and tirzepatide and compared with 46 women on metformin monotherapy (25). Here, a mean change in weight of −8.65 kg (SD, 6.9), a mean change of −0.078 kg/m^2^ in BMI, and a mean percentage weight change of −8.07% (SD, 6.21) were reported in the combination group; the duration of follow-up was not clear (25). In the recent systematic review and meta-analysis by Bo et al comprising 1476 participants from 29 randomized controlled trials (RCTs), combinations of GLP-1 receptor agonists with “standard therapy” reduced body weight (mean difference, −3.44 kg; 95% CI, −6.20 to −0.67), BMI (mean difference, −2.05; 95% CI, −3.55 to −0.55), and waist circumference (mean difference, −4.39 cm; 95% CI, −6.75 to −2.02) (9). No data regarding tirzepatide were included. Other recent systematic reviews and meta-analyses of other antiobesity medications in women with PCOS have either included only 176 women (semaglutide or liraglutide) from 4 RCTs (26) or not been able to perform meta-analytical comparisons for GLP-1 receptor agonist medications and stated that research on the effectiveness of these medications specifically in PCOS is of high priority (15). In the meta-analysis of de Hollanda Morais et al, data from 176 women treated with semaglutide or liraglutide were summarized (26). These GLP-1 RAs were associated with a mean reduction in waist circumference of 5.16 cm (95% CI, 4.21 cm to 6.11 cm), a mean reduction in BMI of 2.42 (95% CI, 1.74 to 3.10), and significant reductions in serum triglycerides and total testosterone (26). A more recent systematic review with meta-analysis of outcomes from liraglutide included data from 330 women from 7 RCTs, reporting significant improvements in menstrual frequency, reduced BMI, and improved insulin resistance based on a random effects model-pooled Hedge's *g* (therefore, a direct comparison with the present study's results is not possible) (27). The present study's sample of over 4000 women with (self-reported) PCOS enrolled in a weight management service with coverage across the United Kingdom provides an important contribution to the evidence by (1) specifically characterizing the real-world effectiveness of tirzepatide in an underresearched group with high unmet clinical need and (2) identifying potential opportunities for augmenting outcomes with nonpharmacological adjuncts.

The strengths of this study include the inclusion of women from a service with national coverage and the exploration of possible modifiers of pharmacological effectiveness. Several limitations must be acknowledged. First, the retrospective observational nature precludes any causal inferences regarding the effects of nonpharmacological adjuncts to tirzepatide. Second, due to its use of a digital weight management service, the study did not routinely capture coded structured information on reproductive, hormonal, or metabolic outcomes beyond weight, precluding assessment of whether tirzepatide could be associated with improvements in ovulatory function, androgen profiles, or PCOS symptomatology. Third, engagement was self-selected and not randomized, introducing the possibility of confounding by motivation, baseline health status, dietary change, education level, varying proclivity to adhere to medication, or other unmeasured confounders. Moreover, reliance on self-reported weight entries introduces potential for both random and systematic measurement error, which could compromise precision and accuracy. Participants may misread or misrecord their weight, use uncalibrated scales, or underreport values due to social desirability, particularly if progress stalls. The robustness of self-reported weight data has been studied elsewhere, particularly with regard to agreement with clinical measurement. Typically, adults overestimate height and underestimate weight, with Sutton et al estimating an average 1 kg/m^2^ discrepancy in BMI when using self-reported data (28). The present study focused on weight change over time; in a recent literature review (29), “higher quality” studies using data from the United States found no changes over time regarding the magnitude of difference between self-reported and measured mean height, weight, and BMI. Therefore, while it is important to recognize the limitations of self-reported weight, we believe that the broad primary finding (that weight loss trajectories in women with and without self-reported PCOS appear similar) is likely to be valid. There may be misclassification bias in the use of self-reported PCOS status; this could be bidirectional (ie, reflecting the increasingly understood underdiagnosis of PCOS in the population) (6, 30, 31) or due to complexities around “correct/appropriate diagnoses” (32, 33). Self-reported PCOS is commonly used in observational and epidemiological studies due to the challenges of obtaining clinical and laboratory assessments. It has been estimated that 82.5% of women with self-reported PCOS based on hirsutism and irregular menses meet the Rotterdam criteria for PCOS when clinically assessed and that PCOS self-report may have a sensitivity of 88% and a specificity of 78% (34). Due to the data source for the present study being from a digital weight management service, detailed clinical and biochemical information regarding diagnoses such as PCOS was not available. Future studies should consider collecting structured data on requisite markers to establish a formal PCOS diagnosis and low-friction methods to collect temporal data on PCOS symptomatology so that weight loss can be robustly correlated with PCOS features over time. Lastly, due to a combination of censoring and attrition occurring during the open-cohort design, the completer analysis was limited in terms of sample size; we plan to update our analyses as follow-up data mature for women using this relatively recently introduced weight management medication.

In conclusion, the findings of this study suggest that tirzepatide may have significant potential for weight management in women with PCOS and provide motivation for future research, particularly in women with biochemical/clinical confirmation of PCOS status. Randomized studies with protracted follow-up and broader data collection could interrogate tirzepatide's effects on insulin resistance, dyslipidemia, androgen profiles, quality of life, and symptomatology (eg, menstrual abnormalities); examine correlations between the extent of weight loss and impacts on cardiometabolic health; and ascertain the sustainability of weight loss in the long term. Such studies should also compare outcomes between “medication-only” and “medication plus nonpharmacological adjuncts,” which will elucidate the scope for augmentation of weight loss beyond pharmacotherapy alone. The impacts of the use of concomitant medications for PCOS should also be considered. Future work should also explore differential responses by PCOS phenotype, ethnicity, and baseline metabolic risk to inform more personalized approaches.

## Data Availability

Restrictions apply to the availability of some or all data generated or analyzed during this study to preserve patient confidentiality or because they were used under license. The corresponding author will, on request, detail the restrictions and any conditions under which access to some data may be provided.

## Presentation

An abstract based on this work was presented as an oral presentation at the Obesity Week 2025 Conference (Atlanta, GA, USA, November 4-7, 2025).
